# Dr. Stokes on Softening of the Heart, &c.

**Published:** 1842-06-04

**Authors:** 


*Softening of the Heart, with thinning of its Parietes.*—Dr. Stokes said that the heart before him was taken from an individual whose case possessed more than ordinary interest, from the great doubt and difference of opinion to which it gave rise. He would detail briefly what he knew of the history of the case. It was an example of that class of affections which every practitioner must have met with at some time or other, in which the diagnosis remains obscure, although most of the symptoms are of an important character. The case he was about to detail would furnish an example. An individual, who appears to be in good health, looks well, has a good appetite, is strong, able to take exercise, and to fulfil the duties of an active profession, applies for medical advice, and the only thing remarkable about him is a singularly irregular and rapid pulse, so quick and so deficient in regularity that it is almost impossible to count it. The action of the heart is also irregular and cannot be analyzed. Of this description of case Dr. Stokes said that every practical man must have seen examples. Looking to the specimen before him it would be hard to define what was its exact pathological condition. The person from whom it was taken had the peculiarity of circulation already mentioned, for several years. Some years since he left this country for a warm climate, and was at that period in perfect health. After residing there for seven or eight years, he was attacked with one of those affections incidental to warm climates, and consulted a military medical officer, who, having remarked the singular state of the circulation, was informed by the patient that he had noticed this condition a long time before; soon after this he was invalided and sent home. He arrived in this country somewhat reduced in strength, but in a short time recovered, with the exception of some cough and difficulty of breathing. He consulted a very eminent medical man, who gave it as his opinion that it was a case of dislocation of the heart consequent on empyema of the left side. He then went to London, where a different opinion was given (the exact nature of which Dr. Stokes did not know,) and after some time returned again to Dublin. When seen by Dr. Stokes he had the appearance of a man in good health. He complained of a cough and difficulty of breathing, particularly on making any active exertion, but stated that he could go about and enjoy society. There was one peculiarity in this case worthy of attention : he stated that he was always better when he took a free allowance of wine, and that abstinence or depleting medicines made him worse. He had no anascara, nor was there any thing in his countenance expressive of disease of the heart. The pulse as before described, the action of the heart quick and irregular, but feeble, and the sound on percussion over the præcordial region not remarkably dull. There was no evidence of effusion into the pleura, and his cough was merely a slight dry catarrh. Dr. Stokes recommended him to go down to the country, and remain there for some time. During his stay there he contracted a bad bronchitis, and let it run on for a week without using any remedy. He returned to Dublin, and when seen by Dr. Stokes, he had violent bronchitis, with sonorous and wheezing rales anteriorly ; posteriorly in both lungs, towards the lower parts, an intense muco-crepitating rale was heard. The action of the heart now became more feeble and irregular, while the same rapid action continued ; and the pulse grew daily weaker and fainter, until it could no longer be felt at the wrist ; and his limbs became œdematous, blue, and cold. The impulse of the heart became gradually weaker and weaker, until at last it appeared to cease altogether. He remained in this state for four days, and then died. Every kind of stimulant was employed with the view of arousing the action of the heart, but without effect. In one night he used a bottle of brandy, and a considerable quantity of wine. The misery he endured from an overwhelming sense of exhaustion appeared to be extreme ; and as his voice, which was naturally strong, never failed him until a few moments before death, his out-cries were truly heart-rending. The symptoms connected with the heart were, extreme irregularity, and extreme rapidity of action, absence of murmur, and very feeble impulse. On opening the thorax the heart appeared to be greatly enlarged ; but this arose from the distension of the right ventricle and auricle, which were very much enlarged and filled with blood. There was no trace of inflammation anywhere. The right ventricle was thin, and appeared to contain more adipose substance than usual, but was not in the fatty condition described by Mr. Smith at a former meeting. The left ventricle was enlarged but not thickened. The whole substance of the heart was softened ; so much so, that it broke down readily under the finger ; and in taking the organ out of the thorax, a rent occurred, passing from the posterior part of the right ventricle across the septum cordis. All the valves were healthy. Dr. Stokes observed that this case would throw some light on the general nature of the disease. There was one point to which he would allude briefly : Laennec states that clearness of sound in affections of the heart indicates dilated cavities ; without denying this, Dr. Stokes would remark that clearness of sound indicated not merely dilatation of cavities or mechanical thinning, but also strong action of the muscular fibres of the organ ; dilatation and thinning might occur without increased clearness of sound.—*Dublin Journal of Medical Science.*

